# The Effects of Family and School Interpersonal Relationships on Depression in Chinese Elementary School Children: The Mediating Role of Academic Stress and the Moderating Role of Self-Esteem

**DOI:** 10.3390/children11030327

**Published:** 2024-03-09

**Authors:** Jinqian Liao, Shuai Chen, Yanling Liu, Cheng Guo

**Affiliations:** 1 Research Center of Mental Health Education, Faculty of Psychology, Southwest University, Chongqing 400715, China; 1155171960@link.cuhk.edu.hk (J.L.); ssq@swu.edu.cn (Y.L.); 2 School of Psychology & Center for Studies of Psychological Application, South China Normal University, Guangzhou 510631, China; chenshuai@m.scnu.edu.cn

**Keywords:** interpersonal relationships, depression, academic stress, self-esteem, elementary school children

## Abstract

This study explores the relative contributions and psychological mechanisms of the effects of family (father–child and mother–child) and school (teacher–student and student–student) interpersonal relationships on depression in elementary school children. The participants (*n* = 20,629) were elementary school children (48.19% male) aged nine to 13 years from Southwest China during the COVID-19 pandemic. They voluntarily completed questionnaires regarding parent–child, teacher–student, and student–student relationships, as well as academic stress and self-esteem. The results indicate that the effect of family interpersonal relationships on children’s depression was stronger than that of school interpersonal relationships. The predictive effects of father–child and mother–child relationships on children’s depression did not significantly differ; however, the effect of student–student relationships was significantly stronger than that of teacher–student relationships. Academic stress partially mediated the effect of interpersonal relationships on depression in elementary school children. The effects of family interpersonal relationships and academic stress on depression were moderated by self-esteem. These findings underscore the disparities and mechanisms pertaining to the impacts of diverse interpersonal associations on children’s depression, thus signifying significant implications for the advancement of research and intervention strategies aimed at addressing this issue.

## 1. Introduction

People with depression exhibit a variety of symptoms including reduced emotional expression, diminished interest, and somatic discomfort [1]. Depression is a significant predictor of an individual’s mental health and also one of the most common mental health problems in children and adolescents [2,3]. According to estimates by the World Health Organization in 2020, approximately 50% of psychological problems emerge before the age of 14, that is, mostly during the elementary school years. Studies have shown that depression has a significant negative impact on the academic development and problematic behaviors of children and adolescents [4,5]. In recent years, there has been a significant increase in research revealing that mental health problems tend to emerge at a young age. A child’s time at elementary school is a critical period in the development of socialization of individuals and is a stage of extensive change in social relationships [6]. Children in elementary school are not yet psychologically mature and are vulnerable to frustration and depression when adapting to new environments or facing stress [7,8]. A meta-analysis that included the detection rate of psychological problems among elementary school students in mainland China from 2010 to 2020 found that the detection rate of depressive symptoms among elementary school students was 14.6% and increased over the years [9]. The outbreak and persistence of COVID-19 posed a further threat to the mental health of children and adolescents. Adolescents who are exposed to unstable environments over the long term tend to experience higher levels of anxiety, greatly increasing the risk of depression among adolescents [10]. A global meta-analysis showed that the prevalence of depression among children and adolescents during the pandemic was 25.2%, significantly higher than the pre-epidemic prevalence, which was 12.9% [11]. Meanwhile, the results of a large-sample survey in China also revealed that the detection rate of depressive symptoms was relatively high in both primary and secondary school students during the pandemic, with elementary school students having a detection rate of 17.3% [12]. Considering the harmful effects and high prevalence of depressive symptoms in childhood, it appears timely to explore the influential factors and the action mechanisms of depression in depth.

Both ecological systems theory [13] and developmental contextualism [14] have revealed that humans inhabit various interconnected and interacting environmental systems, and individuals develop by engaging with the environment and others in each system. As children develop, their living environment gradually expands from being in their single-family unit to include the school, with both family and school being the two most influential microsystems on their overall development. Consequently, children develop and grow as they interact with key members of their family and school communities; thus, the onset of depression in children is closely associated with both environments. Family interpersonal relationships (father–child and mother–child relationships) [15,16] and school interpersonal relationships (teacher–student and student–student relationships) are core components of the family and school microsystems [17,18,19] and have been widely shown to be strongly associated with depressive symptoms in children. However, past research has often only focused on exploring the influence of interpersonal relationships in specific environments on depression or solely examined the role of individual interpersonal factors in the process of depression, while neglecting the comparative study of the two crucial environments in adolescent development: family and school, along with the important interpersonal relationships within these environments. Additionally, previous studies have not been comprehensive enough in comparing the effects of different interpersonal relationships, such as between mother–child and father–child or between teacher–student and student–student, on depression. Although a few studies have noted the different roles of teachers and peers in child development and investigated their effects on child depressive symptoms, there is still a lack of thorough differentiation and comparison of the impact of different interpersonal relationships on depressive emotions in various environments. As research continues, it is increasingly common to examine the shared impact of various contexts on individual development, as well as the distinct effects of different environments from a systematic perspective [20,21,22]. Therefore, the present study integrates different interpersonal relationships in family and school settings under the same research framework to compare in depth their common mechanisms of action and processes of influence on children. This provides theoretical and empirical support for an in-depth understanding of the emergence of depressive symptoms in elementary school children and has important theoretical and practical implications for the prevention and intervention of depression in children.

### 1.1. The Relationship between School Interpersonal Relationships and Depression in Elementary School Students

Theoretical and empirical research has generally shown that interpersonal factors are among the strongest predictors of the onset and persistence of depression [23,24]. Children easily tend to feel acceptance and recognition when they have secure interrelationships with others, which reduces the likelihood of experiencing anxiety and depression. Conversely, interpersonal relationships that lack stability and support may reduce a child’s ability to achieve satisfactory levels of well-being, which can lead to mental health problems [25,26]. For a child’s socialization, the family and school are the two central microsystems in which interpersonal relationships are formed [27]. In the family environment, the parent–child relationship, consisting of a father–child relationship and a mother–child relationship, is a key component of the family system. The parent–child relationship refers to the mutual relationship between parents and children, which is the most important social relationship for children and has significant impacts on their social adaptation and psychological well-being [28,29]. Numerous studies have shown that high-quality parent–child relationships can reduce negative emotions such as anxiety and depression in adolescents and mitigate mental health problems in general [30,31]. Conversely, lower levels of parent–child relationships and attachment represent risk factors for the development and progression of depressive symptoms in children [32,33], which can significantly increase a child’s risk of emotional disturbance [34]. Similarly, in the school environment, interpersonal relationships, consisting of teacher–student and student–student relationships, are also closely related to the psychological and behavioral development of adolescents [35]. A positive school atmosphere and high levels of school connectedness are significantly negatively correlated with depression and stress among Chinese adolescents, while being significantly positively correlated with their sense of happiness [36]. Conversely, poor school interpersonal relationships, such as experiencing campus exclusion, bullying, or victimization, significantly increase individual depressive symptoms [37]. Therefore, we emphasize that interpersonal relationships in both family and school environments play a crucial role in the occurrence of depression and psychological well-being among children [27].

According to ecological systems theory, children’s social networks gradually expand as they develop. Their main scene of life shifts from family to school, with the role of peer relationships becoming increasingly prominent in this process. Therefore, there may be differences in the magnitude of the effects of family and school environments on children’s depression due to the increasing influence of peer relationships over time [13,38]; however, the importance of parents and teachers does not diminish as the importance of peers increases [39]. Numerous studies have shown that positive interpersonal relationships can provide individuals with more social support. Good interpersonal relationships within both the family and school settings significantly influence the positive effects of social support on children. Moreover, this social support can further enhance the stability and intimacy of interpersonal relationships [40]. Some studies suggest that familial support has a greater effect on reducing depression, while teacher or peer support has a relatively small or insignificant effect [41,42,43]. Rueger et al. conducted a large-sample meta-analytic study and found that the impact of support from the family on depression is significantly greater than that of support from teachers [40]. A large body of research on attachment relationships suggests that the basis for positive relationships with peers is grounded by secure attachment to parents [44] and that parental support remains an important indicator of a child’s psychological well-being. A child’s early attachments greatly predict and influence their later adjustment to school life [45], with a lack of parental support more significantly predicting their likelihood of developing depression [43].

Considering the theoretical and empirical research presented above, we propose the following hypothesis:

**H1.**  *Family interpersonal relationships and school interpersonal relationships are negatively correlated with depression in elementary school students, with family interpersonal relationships having a stronger effect on depression than school interpersonal relationships*.

#### 1.1.1. The Relationship between Family Interpersonal Relationships and Depression in Elementary School Students

In addition to considering the relative contributions of interpersonal relationships in different environments, it is necessary to focus on the differences in the roles of various interpersonal relationships in the same environment in the developmental process of children. The family is considered to be the primary socialization medium during childhood, providing the social context in which interactions with family members shape the development, adjustment, and functioning of children and adolescents [29]. Given the differences in the roles and functions played by fathers and mothers in child development, researchers have examined the magnitude of their effects on depressive symptoms in children. However, there is some debate within academia regarding the magnitude of the impact of mother–child and father–child relationships on children’s depressive symptoms [32,33]. The specificity hypothesis suggests that both father–child and mother–child relationships have unique effects on child development and that there is no hierarchy of priorities. Sheeber et al. found that father–child and mother–child relationships were linked to depression in roughly the same way [46]. A recent Chinese survey also showed that although the overall level of father–child attachment was lower than that of mother–child attachment, there was no significant difference in the extent to which the two affected depression in children [47]. This “parenting similarity hypothesis” is supported by the results of a recent large-sample meta-analysis by Rueger et al. in which cross-sectional and longitudinal structures consistently showed similar effects of maternal and paternal support on depression [48]. In conclusion, we propose the following hypothesis:

**H2.**  *Father–child and mother–child relationships in the family are negatively correlated with depression in elementary school students, with both having the same predictive effect on depression in elementary school students*.

#### 1.1.2. The Relationship between School Interpersonal Relationships and Depression in Elementary School Students

As children grow older, their living environment expands to the schoolyard, where school interpersonal relationships mainly comprise teacher–student relationships and student–student relationships [13,36]. Children actively develop and establish connections with their teachers and peers, forming emotional and cognitive bonds with them [35]. Teacher–student relationships are fundamental interpersonal relationships in schools and are key factors influencing students’ psychological well-being and problematic behaviors [18]. Peer relationships primarily refer to interpersonal relationships established and developed among individuals of the same age or similar psychological developmental levels during interactions [18]. High-quality peer relationships can promote prosocial behavior and are important factors influencing children’s socialization [49]. Warm and supportive teacher–student and student–student relationships in the school environment have generally been shown to have an overall positive effect on children’s mental health [50], but there may still be differences in their impact on the development of depression in elementary school students. Although some studies have shown that teacher–student relationships have a greater effect than student–student relationships on depression and overall internalizing problems in high school students [20,21], more studies have found that student–student relationships have a greater impact on students’ depressive symptoms than teacher–student relationships [51]. One longitudinal study revealed that the degree of peer acceptance significantly predicted the joint developmental trajectory of depression and self-injury in adolescents [52]. One diary study also revealed that negative peer interpersonal events uniquely explained the persistence of depressive symptoms [53]. Critically, the results of Rueger et al.’s meta-analysis also showed that peer support was significantly more predictive of depression in children and adolescents than teacher influence and comparable to the role of family [40]. Therefore, combining the results of the above studies, we propose the following hypothesis:

**H3.**  *Teacher–student and student–student relationships in the school are negatively correlated with depression in elementary school students, with student–student relationships having a stronger effect on depression than teacher–student relationships*.

### 1.2. The Mediating Effect of Academic Stress

Beyond the effects of different interpersonal relationships on childhood depression, psychological mechanisms also affect depression, and these can help provide a deeper understanding. Zuroff et al. proposed a more comprehensive dynamic interactionist model of vulnerability to depression [54] by integrating person–context interaction theory [55] and the theory of depressive self-schemas [56] into traditional theories on vulnerability to depression. This model comprehensively highlights the influence of interpersonal relationships, individual vulnerability, and stressful events on depression in the environment. The dynamic interactionist model of vulnerability to depression [54] suggests that poor interpersonal relationships may lead to an individual’s depression by generating stressful events. Given that academic stress negatively affects children and adolescents [57] and is one of the most significant sources of stress for them, we argue that it may play a central role in causing depression. Academic stress is often defined as the psychological pressure resulting from school affairs, e.g., examination results, academic competition with peers, and academic expectations from parents and teachers [58]. Excessive academic stress can negatively impact students’ physical health [59], mental health [60], and academic development [61,62,63]. Research has found that secondary school students have been in a continuous state of panic since the outbreak of the epidemic. Additionally, prolonged periods of home isolation coupled with a lack of exercise have led to increased academic pressure and below-average health conditions [10,12]. The results of numerous cross-sectional and longitudinal studies have confirmed that that academic stress is significantly and positively associated with depressive symptoms [3,18,64,65,66,67].

Based on the theory of depressive self-schemas [56], the social support that students perceive from their families and schools can support them in resolving stressors in their learning process, thus reducing their risk of developing depressive symptoms [68]. Specifically, in the family and school environment, positive parenting styles, a favorable environmental climate, belongingness to the campus, and close interpersonal relationships have not only significantly predicted levels of academic stress [48,59,65], but also correlated closely with an individual’s overall perception and regulation of stress [67,68,69,70,71,72]. Based on the above theoretical and empirical findings, we propose the following hypothesis:

**H4.**  *Academic stress plays a mediating role in the effect of family interpersonal relationships and school interpersonal relationships regarding depression in elementary school students*.

### 1.3. The Moderating Effect of Self-Esteem

Finally, the effects and processes of interpersonal relationships can vary depending on a child’s own traits. Zuroff et al. [54] further noted that poor interpersonal relationships not only directly contribute to depression, but also generate specific and externalized stressful events, which in turn act in conjunction with individual vulnerability factors leading to depression. Among the various traditional theoretical models of depression, low self-esteem has been consistently identified as a key susceptibility factor playing a decisive role in the onset and maintenance of depression. Self-esteem, a core component of the ego system, concerns an individual’s overall attitude, evaluation, and beliefs about their self and self-worth [73]. Having high self-esteem has positive implications for an individual’s physical and mental health, academic work, and interpersonal relationships and can alleviate the effects of stressful life events on depression [74,75], while having low self-esteem is a risk factor for various types of problematic behaviors and psychological disorders [76,77]. The relationship between self-esteem and depression has also been at the core of psychologists’ attention for decades. Numerous empirical studies and longitudinal meta-analyses have also revealed that high self-esteem is significantly and negatively related to an individual’s level of depression and that at an early age, the level of self-esteem predicts subsequent levels of depression [76,78,79].

Person–context interaction theory suggests that individual factors interact with environmental factors to influence individual development, which provides theoretical support for the effect of self-esteem-moderating interpersonal relationships regarding depression [80]. Both empirical research studies of Lu et al. and Dang et al. suggested that high self-esteem significantly moderated the effects of parent–child, teacher–student, and same-sex student–student relationships on depression [20,81]; i.e., the negative predictive effect of interpersonal relationships and depression is smaller among children with high self-esteem. A series of vulnerability–stress models [54,82] indicated that the interaction of an individual’s presence of a vulnerability factor (low self-esteem) with an encountered stressful event (academic stress) can play a major role in the triggering and maintenance of an individual’s depressive disorder. The self-esteem alleviation hypothesis and empirical research [74,75,83] further specify that individuals with high self-esteem have more psychological resources for coping better with stressful events and thus, avoid depression often caused by them. Based on the above, self-esteem plays an important role in the triggering and maintenance of depression, so we propose the following hypotheses:

**H5.**  *Self-esteem plays a moderating role in the effects of family interpersonal relationships and school interpersonal relationships on depression in elementary school children. The effects of family interpersonal relationships and school interpersonal relationships on depression are weaker in children with high self-esteem relative to children with low self-esteem*.

**H6.**  *Self-esteem plays a moderating role in the effects of academic stress on depression in elementary school children. The effects of academic stress on depression are weaker in children with high self-esteem relative to children with low self-esteem*.

### 1.4. The Present Study

This study was conducted under the guidance of a theoretical framework that integrates ecological systems theory, the dynamic interactionist model of vulnerability to depression, and person–context interaction theory.

**Figure 1:**
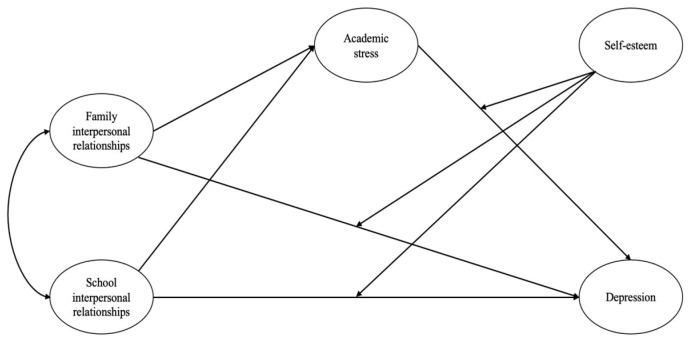
Hypothesis model.

The first aim of this study was to examine the effects and magnitude of interpersonal relationships (family interpersonal relationships vs. school interpersonal relationships) in different environments and different interpersonal relationships (family interpersonal relationships, i.e., father–child vs. mother–child relationships; school interpersonal relationships, i.e., teacher–student vs. student–student relationships), in the same environment, on depression in elementary school students. The second aim was to construct a moderated mediation model to examine the mediating role of academic stress between family and school interpersonal relationships and depression in elementary school students, as well as the moderating role of self-esteem in this mediating process. This study integrates three key dimensions—interpersonal relationships, stressful events, and vulnerability factors—to reveal the causes of depression in children and the links between each system to intervene and prevent depression in children (see Figure 1 for the hypothetical model).

## 2. Materials and Methods

### 2.1. Participants and Procedure

In September 2022, a voluntary, uniform online questionnaire was administered to 21,469 students in Grades 4 to 6 from 22 elementary schools in Sichuan and Chongqing, China, by using whole-group convenience sampling. Invalid subjects were excluded based on authenticity screenings; the final valid responses totaled 20,629 (M = 10.72, SD = 10.96, 48.19% male), with an effective return rate of 96.09%. The demographic information of participants is shown in Table 1.

### 2.2. Measures

#### 2.2.1. Demographic Characteristics

The five demographic measures were the following: gender, age, grade level, only child or not, and parents’ education level.

#### 2.2.2. Family Interpersonal Relationships (Father–Child Relationship, Mother–Child Relationship)

The family interpersonal relationship was measured using the Cohesion Evaluation subscales of the Family Adaption and Cohesion Evaluation Scales [84], including two subscales with the same questions, father–child and mother–child affinity, with 10 items each (e.g., “I feel very close to my father/mother”). Participants responded using a five-point Likert scale that measured the frequency (1 = never to 5 = always) of feelings. The mean scores of all items were calculated, with higher scores representing higher levels of the parent–child relationship, implying a stronger emotional connection between the individuals and their fathers/mothers. The internal consistency of the father–child subscale was 0.77 (Cronbach′s α) while the mother–child subscale was 0.75. Cronbach′s α of the full questionnaire was 0.85.

#### 2.2.3. School Interpersonal Relationships (Teacher–Student Relationships, Student Relationships)

School interpersonal relationships were measured using the Delaware School Climate Survey-Student (DSCS-S) [85]. The teacher–student relationship (“teachers like their students”) and student–student relationship (“students respect each other”) subscales of this scale were used, each consisting of five items. Each item was scored from 1 (full disagreement) to 5 (full agreement). The mean scores of all items were calculated with high scores representing higher levels of teacher–student and student relationships as perceived in school. Cronbach′s α of the teacher–student relationship subscale was 0.93 and the student relationship subscale was 0.96. Cronbach′s α of the full questionnaire was 0.95.

#### 2.2.4. Academic Stress

Academic stress was measured by the Academic Stress subscale of the Adolescent Self-Rating Life Events Checklist [57], which consists of five stress factors (e.g., “heavy academic stress burden”). Participants responded using a six-point Likert scale that evaluated the frequency from 0 (never happened) to 5 (happened with extremely heavy impact). The higher the mean scores were on all items, the higher the academic stress was of the individual. Cronbach’s α was 0.81 for this scale.

#### 2.2.5. Self-Esteem

The Rosenberg Self-Esteem Scale (RSES) [73] revised by P. Wang et al. was adopted [86]; it included 10 questions (e.g., “I feel I have many good qualities”). Responses were rated on a four-point scale, ranging from 1 (strongly disagree) to 4 (strongly agree). The mean scores of all items were calculated to represent the level of self-esteem. Cronbach’s α was 0.86 for this scale.

#### 2.2.6. Depression

The Patient Health Questionnaire-9 (PHQ-9) [87] was used to examine the level of the participants’ depression. The scale is a nine-item self-assessment tool (e.g., “Feeling down, depressed, or hopeless”) based on major depressive disorders (MDDs) of the Diagnostic and Statistical Manual of Mental Disorders Fourth Edition (DSM-IV). Responses were measured on a four-point scale ranging from 1 (never) to 5 (nearly every day). A high mean score on all the questions indicated a high level of depression. Cronbach’s α was 0.89 for this scale.

### 2.3. Research Procedures and Data Analysis Strategy

All data were collected and downloaded online through Questionnaire Star. All participants gave informed consent and voluntarily completed the online questionnaire with the knowledge and cooperation of school authorities and the participants’ parents. SPSS 25.0 and AMOS 24.0 were used to analyze the data. The analysis steps were the following:

(1) The Harman single-factor test was used to examine the common method bias, while descriptive statistics and Pearson’s correlation analysis were used to test the mean, standard deviation, correlation coefficient, skewness, and kurtosis of the study variables.
(2) We employed the restricted model method used in previous research [21,47] to compare the magnitude of the effect of family and school interpersonal relationships on the participants’ level of depression. Additionally, we compared the impact of father–child and mother–child relationships in the family environment and the effect of teacher–student and student relationships in the school environment on children’s depression.
(3) A two-step approach was employed to validate the mediating role of academic stress. First, a structural equation model was developed to investigate the relationship between the independent variable (school interpersonal relationships and family interpersonal relationships) and the dependent variable (depression in elementary school children), and in the second step, a structural equation model was constructed including the mediating variable (academic stress). In the nonparametric percentile bootstrap method, with 5000 repetitions for bias correction, mediation effect testing and confidence interval estimation were conducted. A significant mediation effect was indicated if the 95% confidence interval did not contain zero.
(4) The product index method was used to construct the interaction term and test the moderated mediating effect. The significant predictive role of interaction terms indicated the presence of a significant moderation effect, and simple slope analysis was conducted to determine the moderation effect pattern.

The maximum likelihood method was used to estimate the model parameters. The goodness-of-fit of the model was assessed using the Chi-square, GFI (≥0.90), CFI (≥0.90), TLI (≥0.90), RMSEA (≤0.05 excellent, ≤0.080 acceptable), and SRMR (≤0.05 excellent. *p* < 0.05 denotes statistical significance. Gender, grade level, only child status, and average parental education were included as control variables in each analysis.

## 3. Results

### 3.1. Testing for Common Method Bias

The Harman single-factor method was used to test for common method bias resulting in the extraction of 10 factors with eigenvalues greater than 1. The first factor had an explanatory power of 26.28%, which is below the critical threshold of 40%. Therefore, this result did not suffer from significant common method bias.

### 3.2. Descriptive Statistics and Correlation Analysis

Table 2 presents the mean, standard deviation, and correlation matrix for each variable. The results show that there are significant positive correlations between school interpersonal relationships (teacher–student and student relationships) and family interpersonal relationships (father–child and mother–child relationships) with self-esteem and significant negative correlations with academic stress and depression. Self-esteem shows significant negative correlations with academic stress and depression, while academic stress is significantly positively correlated with depression. Additionally, the absolute values of kurtosis (0.02 < |Kurtosis| < 4.47) for each variable are less than 10, and the absolute values of skewness (0.27 < |Skewness| < 1.96) are less than 3, indicating approximate normality of the data. Consequently, the results of the descriptive statistics, correlation analysis, and tests of data normality support the subsequent analysis of the structural equation model.

### 3.3. Testing and Comparing the Effects of Family and School Interpersonal Relationships on Depression in Children

We used structural equation modeling to analyze the relationships between variables. First, we compared the effects and differences in interpersonal relationships within the family and school environments on childhood depression. Gender, grade level, only child status, and average parental education level were controlled as covariates. The overall fit index of the model was good (χ^2^ = 5066.44, *df* = 102, *p* < 0.001, GFI = 0.97, CFI = 0.96, TLI = 0.94, RMSEA = 0.05, SRMR = 0.02). Among these, both family interpersonal relationships (*β* = −0.47, *p* < 0.001) and school interpersonal relationships (*β* = −0.10, *p* < 0.001) were negatively correlated with depression in elementary school students, together explaining 28.34% of the variance in depression. A nested model comparison approach was used to restrict the coefficients of family and school interpersonal relationships to be equal predictors of depression, and the amount of change in the competing model compared to the original model was obtained as Δχ^2^ (1) = 485.15, *p* < 0.001, indicating that the predictive effects of family interpersonal relationships and school interpersonal relationships on depression in children differed significantly, with family interpersonal relationships having a stronger effect on depression in elementary school children than school interpersonal relationships.

Second, we examined and compared the effects and differences in father–child and mother–child relationships in family environments on childhood depression. The overall fit index of the model was good after controlling for irrelevant variables (χ^2^ = 10,730.78, *df* = 135, *p* < 0.001, GFI = 0.95, CFI = 0.93, TLI = 0.91, RMSEA = 0.06, SRMR = 0.03). Father–child relationship (*β* = −0.26, *p* < 0.001) and mother–child relationship (*β* = −0.24, *p* < 0.001) were negatively correlated with depression in children, with both explaining 21.83% of the variance in depression. A nested model comparison approach was used to restrict the coefficients of father–child and mother–child relationships as equal predictors of depression, and the amount of change in the competing model compared to the original model was obtained as Δχ^2^ (1) = 1.369, *p* = 0.24, indicating that the predictive effects of father–child and mother–child relationships on children’s depression are not significantly different, i.e., father–child and mother–child relationships are equally important in influencing depression in elementary school children.

Finally, we examined and compared the effects and differences in teacher–student and student–student relationships in school environments on childhood depression. The overall fit index of the model was good after controlling for irrelevant variables (χ^2^ = 8821.86, *df* = 213, *p* < 0.001, GFI = 0.96, CFI = 0.97, TLI = 0.97, RMSEA = 0.04, SRMR = 0.02). Teacher–student (*β* = −0.05, *p* < 0.001) and student–student relationships (*β* = −0.26, *p* < 0.001) were negatively correlated with depression in children, together explaining 11.21% of the variance in depression. A nested model comparison approach was used to restrict the coefficients of teacher–student and student–student relationships to be equal predictors of depression, and the amount of change in the competing model compared to the original model was obtained as Δχ^2^ (1) = 104.92, *p* < 0.001, indicating that the predictive effects of teacher–student and student relationships on depression in children differed significantly, with the student–student relationship having a stronger effect on depression in elementary school children than the teacher–student relationship.

### 3.4. The Mediating Effect of Academic Stress

**Figure 2:**
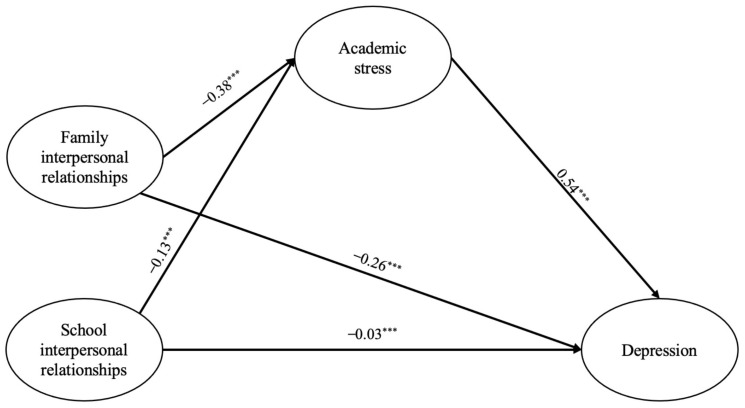
Results of the mediating effect. Note: Indicator and control variables are not shown, *** *p* < 0.001.

Utilizing family and school interpersonal relationships as exogenous latent variables and depression as an endogenous latent variable, academic stress was included as a mediating variable in the structural equation model. The overall fit index of the model was good after controlling for irrelevant variables (χ^2^ = 8157.07, *df* = 185, *p* < 0.001, GFI = 0.96, CFI = 0.95, TLI = 0.94, RMSEA = 0.05, SRMR = 0.03). The results of the model (Figure 2) showed that both family interpersonal relationships (*β* = −0.38, *p* < 0.001) and school interpersonal relationships (*β* = −0.13, *p* < 0.001) were negatively correlated with academic stress, and academic stress (*β* = 0.54, *p* < 0.001) was positively correlated with depression in elementary school students. Furthermore, compared to the direct effects, the predictive effects of both school interpersonal relationships (*β* = −0.03, *p* < 0.001) and family interpersonal relationships (*β* = −0.26, *p* < 0.001) on depression in elementary school children declined after mediation from academic stress, which together explained 22.56% of the variance in academic stress and 51.04% of the variance in depression.

The bootstrap method, with repeated sampling 5000 times, was used to test the mediating role of academic stress. The result revealed a significant mediating effect of academic stress between both family (*β* = −0.21, *p* < 0.001, 95% CI: [−0.22, −0.19]) and school interpersonal relationships (*β* = −0.07, *p* < 0.001, 95% CI: [−0.08, −0.06]) and depression, with the mediating effect accounting for 46.81% (family interpersonal relationships) and 70.00% (school interpersonal relationships) of the total effect, respectively.

### 3.5. The Moderating Effect of Self-Esteem

We used latent variable moderating effect analysis to analyze the moderating role of self-esteem in mediating the effects of academic stress on school and family interpersonal relationships on depression in elementary school students. First, the factor–load balancing method in item-packed measures, recommended by Wu and Wen [88], was used to package self-esteem and academic stress into two indicators each. Consistent with prior research, family interpersonal relationships were measured using two indicators, father–child and mother–child relationships, and school interpersonal relationships were also assessed using two indicators, teacher–student and student–student relationships. Depression was measured using nine indicators, all of which were standardized. Next, the indicators were paired and multiplied according to the “large matches large, small matches small” principle to construct the interaction terms of self-esteem and family interpersonal relationship, self-esteem, and school interpersonal relationship, as well as self-esteem and academic stress (two indicators for each). Finally, a structural equation model was constructed and fitted to the data for estimation. The fit indices of the model were χ^2^ = 15,365.36, *df* = 265, *p* < 0.001, GFI = 0.95, CFI = 0.93, TLI = 0.91, RMSEA = 0.05, and SRMR = 0.04, indicating good model fit.

**Figure 3:**
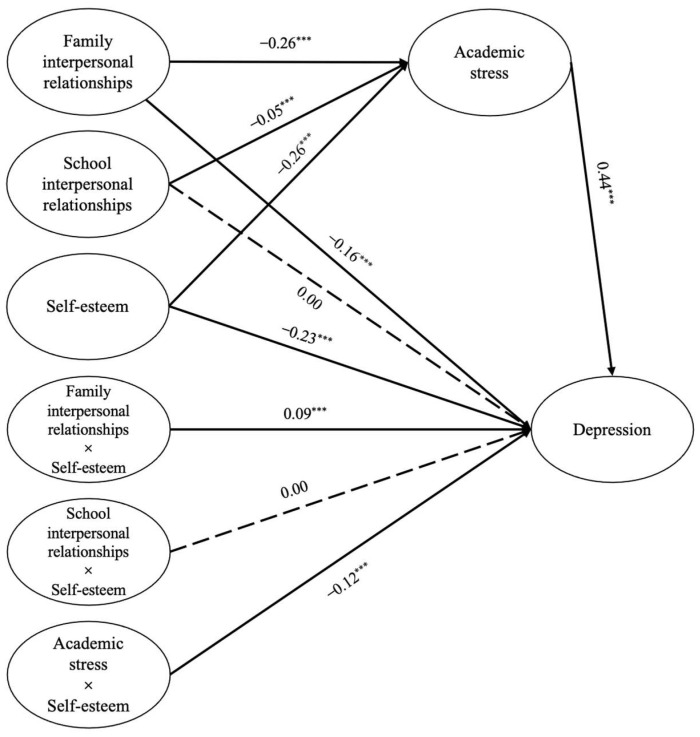
Results of the moderated mediation analysis. Note: Indicator and control variables are not shown, *** *p* < 0.001.

The results of the model analysis (Figure 3) showed that family interpersonal relationships (*β* = −0.26, *p* < 0.001), school interpersonal relationships (*β* = −0.05, *p* < 0.001), and self-esteem (*β* = −0.26, *p* < 0.001) were negatively correlated with academic stress, and academic stress was positively correlated with depression in elementary school students (*β* = 0.44, *p* < 0.001), while family interpersonal relationships (*β* = −0.16, *p* < 0.001) and self-esteem (*β* = −0.23, *p* < 0.001) were negatively correlated with depression. The predictive effect of school interpersonal relationships (*β* = 0.00, *p* > 0.05) on depression was no longer significant. Furthermore, the interaction terms between self-esteem and academic stress (*β* = −0.12, *p* < 0.001) were negatively correlated with depression, while the interaction term between self-esteem and family interpersonal relationships (*β* = 0.09, *p* < 0.001) was positively correlated with depression. The interaction term between self-esteem and school interpersonal relationships (*β* = 0.00, *p* > 0.05) on depression was not significant, indicating that the effects of academic stress and family interpersonal relationships on depression were moderated by self-esteem.

**Figure 4:**
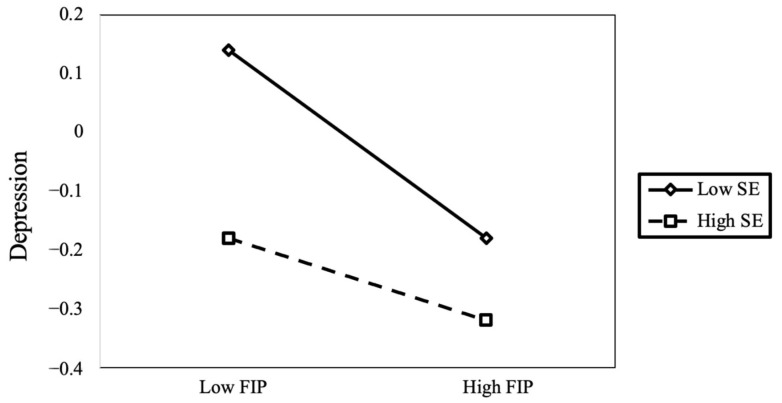
The moderating role of self-esteem in the relationship between family interpersonal relationships and depression.

**Figure 5:**
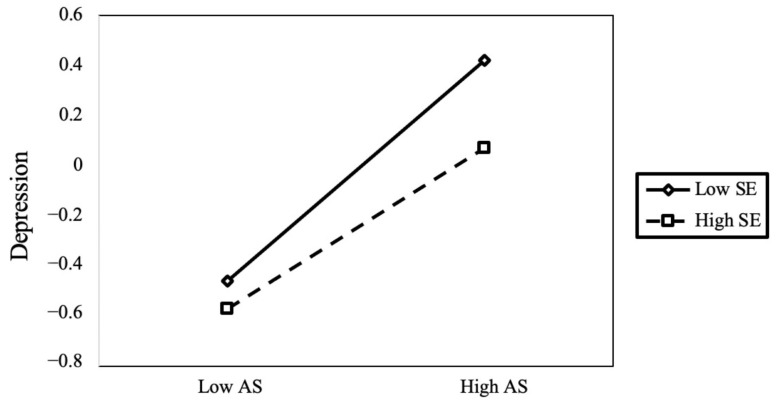
The moderating role of self-esteem in the relationship between academic stress and depression.

To explain the specific pattern of self-esteem regulation effects, we conducted a simple slope analysis using a classification scheme in which individuals scoring one standard deviation above the mean were designated as the high self-esteem group, while those scoring one standard deviation below the mean were designated as the low self-esteem group. The analysis of the moderating effect of self-esteem on the relationship between academic stress and depression (Figure 4) revealed that when self-esteem levels were low, academic stress significantly and positively predicted depression (*B*_simple_ = 0.45, *p* < 0.001, 95% CI = [0.43, 0.48]). Conversely, when self-esteem levels were high, the positive predictive effect of academic stress on depression remained significant but was attenuated (*B*_simple_ = −0.27, *p* < 0.001, 95% CI = [0.24, 0.30]). Furthermore, the analysis of the moderating effect of self-esteem on the relationship between family interpersonal relationships and depression (Figure 5) revealed that when self-esteem levels were low, family interpersonal relationships were negatively correlated with depression (*B*_simple_ = −0.22, *p* < 0.001, 95% CI = [−0.26, −0.19]). Conversely, when self-esteem levels were high, the negative correlation of family interpersonal relationships on depression remained significant but diminished (*B*_simple_ = −0.06, *p* < 0.001, 95% CI = [−0.08, −0.03]).

## 4. Discussion

Given the high prevalence [9,11,12] and long-term detrimental effects of child depression, it is imperative to gain a deeper understanding of the factors that influence the onset of depression in children. Both ecological systems theory and extensive empirical findings [32,40] have highlighted the importance of various interpersonal relationships in family and school environments as factors in child depression. However, there remains significant debate in the academic community as to which interpersonal relationships are more important when considering both different environments and different interpersonal relationships. Thus, the potential mechanisms and conditions under which interpersonal relationships influence depression in children need to be further explored. This study provides new evidence for research in this area by explaining the pattern of influence of family and school interpersonal relationships on depression in elementary school children and by verifying the mediating role of academic stress and the moderating role of self-esteem.

### 4.1. Interpersonal Relationships and Depression in Elementary School Students

We found that both family (including father–child and mother–child relationships) and school (including teacher–student and student–student relationships) interpersonal relationships were negatively correlated with depression in children, which supports H1 and is fully consistent with many theories [23,55,56] and empirical studies [30,31,89] on depression. On the one hand, positive family and school relationships are the strongest social support for children, providing them protection against depression. Also, self-determination theory [90] states that individuals develop and build a sense of security for themselves and others when the social environment satisfies their psychological need for relatedness as a result of the interaction and development between the individual’s psychological needs (i.e., autonomy, relationships, and competence) and the social environment. In other words, positive interpersonal experiences satisfy children’s basic psychological needs and lead to increased personal growth and well-being, thus reducing the probability of depressive symptoms [91]. On the other hand, good interpersonal relationships allow children to develop positive cognitive schemas and self-evaluations, which reduce their risk of depression. According to attachment theory, during interactions with principal members, children develop internal working models of self, others, and relationships (family and school), and secure and stable attachment relationships exhibit positive models of self (valuable and worthy of love) and others (responsive and trustworthy), thus ensuring their healthy development. Conversely, children with insecure attachment relationships exhibit negative representations of self and others and develop a range of negative affective, cognitive, and behavioral styles, which leads to depression-like symptoms [47].

We also found a significantly stronger effect of family interpersonal relationships on children’s depression than school interpersonal relationships, which supports H1 and is consistent with the results of Rueger et al.’s meta-analysis and a growing body of literature [40,41,42,52].

This finding reaffirms the common view that social support from specific sources varies by developmental stage and that the role of family is critical for individuals in both childhood and adolescence [40,92]. China’s social context and its education policies may also account for the prominence of the role of the family. The home isolation policy in the context of COVID-19 increased the time students spent with their parents. Online teaching also limited children’s interaction with teachers and peers, which meant that parents became the most important source of support for children, leading to the role of parents in children’s emotional development becoming more important. In July 2021, China enacted the “Double Reduction Policy” and the “Family Education Promotion Law”, both of which emphasize the need to strengthen family education. As a result, children consider their parents a source of security in their lives [93] and they also receive more guidance from their parents in their academic development, making them more dependent on their parents. The increase in the length of time parents spend with children in their studies helps elementary school students develop better study habits and improve their academic level and sense of self-worth, thus reducing their psychological problems [65,89,91].

We identified the same predictive effect of father–child and mother–child relationships on their children’s depression, which supports H2 and is also consistent with the parenting similarity hypothesis [48] and some of the findings of other studies [46,47]. This finding suggests that the traditional view of fathers as children’s “secondary caregivers” is inconsistent with the facts [94]. Fathers interact with their children in unique ways and play an irreplaceable role in their emotional and social functioning [95]. Fathers spend more time with their children and invest more in their upbringing [96], which is likely to play an increasingly important role in healthy child development.

Notably, student–student relationships appeared to have a greater effect on depression in children than teacher–student relationships in the school environment, which supports H3 aligning with the idea that student–student relationships are more significant [52,53]; however, this finding conflicts with previous studies noting the greater importance of the teacher–student relationship [20,21,42] We hypothesize that this may be related to the social groups in these studies; the group of students at the elementary school level investigated in the present study tended to rely heavily on peer interactions to explore new ideas, experiences, and emotions. Interactions with peers allowed them to learn how to navigate social situations, develop friendships, and form their own identities in a safe and supportive environment. Although teachers are an important source of support, they may not always fully understand the social dynamics and pressures children face, making it difficult for them to provide effective support and guidance in these areas. Interactions with teachers may be perceived by children as more formal and authoritative with limited frequency and depth of interaction, and mostly limited to learning, and therefore, may be perceived as less enjoyable. Children may feel more pressure to conform to expectations and rules in interactions with teachers, while interactions with peers tend to be more casual and relaxed. The meta-analysis by Rueger et al. also showed that the positive effect of family support on children was equal to that of peer-to-peer support and greater than that of teacher–student support [40].

### 4.2. The Mediating Role of Academic Stress and the Moderating Role of Self-Esteem

We also explored the potential mediating and moderating mechanisms by which interpersonal relationships influence depression. We found that academic stress mediates the influence of family and school interpersonal relationships on children’s depression, which is consistent with H4. This result supports the theory of depressive self-schemas and the dynamic interactionist model of vulnerability to depression [54,56], in which negative interpersonal events exacerbate depressive symptoms by generating strong stressors. Specifically, positive, warm, interpersonal relationships allow children to feel more confident in facing the challenges and demands of learning by affirming themselves and generating higher core self-evaluations, thus reducing academic stress. At the same time, supportive relationships enhance children’s mental capacity and resilience for coping with academic challenges and setbacks, all of which diminish feelings of stress [64,68]. Conversely, negative and unsupportive interpersonal relationships reduce children’s ability to cope, which can lead to more academic stress. Additionally, less perceived academic stress contributes to children’s improved academic motivation, academic efficacy, and performance, which reduces emotional problems [61,62]. In contrast, high levels of academic stress can lead children to use more non-adaptive coping strategies, especially excessive use of the Internet to escape reality, which is detrimental to their mental health [97]. In conclusion, positive interpersonal relationships help children cope effectively with academic challenges and reduce their academic stress, thus preventing or alleviating depression.

Self-esteem moderated the mediating process of academic stress in interpersonal relationships abating the potential for child depression. Positive self-esteem can prevent depressive symptoms in children, which indicates that low self-esteem is a factor in depression [77,98]. Positive self-esteem can also alleviate the effects of academic stress and difficult family interpersonal relationships, a fact supported by a range of vulnerability–stress models of depression [54,82], especially the self-esteem alleviation hypothesis [88]. When faced with challenging life situations (high academic stress, poor parent–child relationships), children with high levels of self-esteem have a better coping ability and thus avoid depression. Conversely, children with low self-esteem have less ability to cope and disengage from negative events which can lead to depression. Self-esteem did not moderate the effect of school interpersonal relationships on depression, although studies have questioned whether self-esteem plays a buffering role. Thus, whether self-esteem can moderate the effects of environmental factors on depression seems to vary depending on those factors. This result partially supports H5 and fully supports H6.

### 4.3. Strengths, Limitations, and Future Directions

The findings of our study appear to clarify the effects of different environments and different interpersonal relationships in the same environment on child depression based on ecological systems theory. We explored the potential mechanisms underlying the relationship between interpersonal relationships and depression, namely, the mediating role of academic stress and the moderating role of self-esteem, based on the dynamic interactionist model of vulnerability to depression and person–context interaction theory. These findings provide an in-depth understanding of the role played by interpersonal relationships, which can bring important theoretical insights and practical implications for children suffering from depression. At the theoretical level, we highlighted the differences in the roles of different interpersonal relationships on depression, underscoring the prominent role of family (vs. school) and peers (vs. teachers) in the development of depressive symptoms in children, while demonstrating the equal influence both parents can have on depression in their children. This inspires us to pay more attention to family education and promote its complete and comprehensive positive development. The previous notion that mothers are the primary drivers of family activities and child-rearing is not advisable. Emotional connections between fathers and children also play an important role in child development. Both parents have unique influences on children’s development, and there is no distinction between primary and secondary roles. Therefore, both parents should be aware of and assume their educational responsibilities, avoiding being “missing” in family education. Additionally, the importance of family relationships suggests that parents should not rely solely on schools for educational responsibilities. They should strengthen communication and emotional exchanges with their children, establish positive parent–child relationships, and create a healthy growth environment for their children. The findings also highlight the importance of school education. Since the relationship between children and their peers is more important, schools should create opportunities for students to learn and cooperate with each other to enhance student–student relationships. Further, despite the rather minimal role played by teachers, it is important not to overlook their impact. Teachers should not only actively show care for students but also meet their emotional connections in the school environment. They should be aware of the significant impact of student–student relationships in the school environment and guide students to establish positive interactive relationships with other students.

In contrast to previous studies that have focused on the effects of interpersonal relationships for alleviating depression, we would like to assert that interpersonal relationships can also influence the development of depression through the creation or elimination of stressful events, which supports the dynamic interactionist model of vulnerability to depression [54] and contributes to an understanding of the relationship between interpersonal relationships, stress, and depression from a different perspective. The results of this study also suggest that parents and schools should fully implement China’s “Double Reduction Policy” to protect children’s mental health by reducing academic stress. Finally, we found that children’s self-esteem, which develops gradually, can counteract the process of environmental influences on depression. Therefore, parents and teachers should focus on nurturing and developing children’s self-esteem at home and in school, while identifying children with low self-esteem and providing them with targeted interventions.

Our study is not without its shortcomings. First, as with other cross-sectional studies, despite our solid theoretical foundation, we cannot infer causal relationships among variables because of its cross-sectional design; thus, more studies are needed to verify them. Second, it is difficult to eliminate the social approval effect and the subjectivity of the results when using participants’ single self-reports; future studies may consider using multiple information sources such as other reviews and qualitative methods. Furthermore, the data in this study are limited to a sample of Chinese elementary school students. Hence, there may be certain limitations in generalizing the results to other age groups or geographic populations. To further enhance this study, future research could consider conducting comparative studies across educational stages or between Eastern and Western cultures to explore differences and commonalities among groups in different contexts. Finally, although academic stress was found to be one of the most dominant stressors, the role of other domain-specific stress perceptions in the development of depression in children and adolescents, both in China and beyond, can also be considered.

## 5. Conclusions

Depression is one of the most debilitating illnesses of our time. The fact that it sometimes begins in childhood is especially concerning, making interventions at this early stage particularly important. Our study reveals that social interpersonal relationships play an important role in both the emergence and development of depression in children, with family interpersonal relationships and student–student relationships playing a particularly prominent role. In addition, academic stress mediates the effect of interpersonal relationships on depression in children. This mediating process also appears to be moderated by a child’s own self-esteem levels. Our findings have important theoretical and practical implications highlighting the combined roles of interpersonal relationships, stressful events, and self-esteem in the development of child depression.

## Figures and Tables

**Table 1:** Demographic characteristics of participants.

Characteristic	*N* (%)	Characteristic	*N* (%)	Characteristic	*N* (%)
Sex		Father’s Education Level		Mother’s Education Level	
Male	9942 (48.19%)	1.	779 (3.78%)	1.	851 (4.13%)
Female	10,687 (51.8%)	2.	4431 (21.48%)	2.	4453 (21.59%)
Grade		3.	5667 (27.47%)	3.	5890 (28.55%)
4th	6887 (33.39%)	4.	4508 (21.85%)	4.	4176 (20.24%)
5th	7162 (34.72%)	5.	4389 (21.28%)	5.	4602 (22.3%)
6th	6580 (31.89%)	6.	855 (4.14%)	6.	757 (3.67%)
Only child status					
Only child	9753 (47.28%)				
Non-only child	10,876 (52.72%)				

Note: For the children’s father’s/mother’s education level, “1.” represents elementary school or below, “2.” represents junior high school, “3.” represents senior high school, “4.” represents junior college, “5.” represents a bachelor’s degree, and “6.” represents a postgraduate degree or higher.

**Table 2:** Descriptive statistics and correlation analysis for each variable (*N* = 20,629).

Variable	M	SD	1	2	3	4	5	6	7	8
1. School interpersonal relationships	3.55	0.54	–							
2. Student–student relationship	3.50	0.62	0.92 ***	–						
3. Teacher–student relationship	3.59	0.57	0.91 ***	0.67 ***	–					
4. Family interpersonal relationships	3.92	0.63	0.38 ***	0.35 ***	0.34 ***	–				
5. Father–child relationship	3.79	0.73	0.35 ***	0.33 ***	0.31 ***	0.90 ***	–			
6. Mother–child relationship	4.04	0.67	0.32 ***	0.30 ***	0.30 ***	0.88 ***	0.59 ***	–		
7. Self-esteem	3.25	0.51	0.39 ***	0.37 ***	0.34 ***	0.54 ***	0.49 ***	0.48 ***	–	
8. Academic stress	0.87	0.88	−0.25 ***	−0.25 ***	−0.20 ***	−0.36 ***	−0.33 ***	−0.31 ***	−0.40 ***	–
9. Depression	3.66	4.72	−0.28 ***	−0.28 ***	−0.23 ***	−0.43 ***	−0.39 ***	−0.38 ***	−0.50 ***	0.58 ***

Note: *** *p* < 0.001.

## Data Availability

The data presented in this study are available upon request from the corresponding author. The data are not publicly available due to confidentiality and research ethics.
